# Hemin Augments Growth and Hemoglobinization of Erythroid Precursors from Patients with Diamond-Blackfan Anemia

**DOI:** 10.1155/2012/940260

**Published:** 2012-05-13

**Authors:** Eitan Fibach, Memet Aker

**Affiliations:** ^1^Department of Hematology, Hadassah – Hebrew University Medical Center, Ein-Kerem, Jerusalem 91120, Israel; ^2^Departments of Pediatrics and Bone Marrow Transplantation, Hadassah – Hebrew University Medical Center, Jerusalem 91120, Israel

## Abstract

Diamond-Blackfan anemia (DBA) is congenital pure red-cell anemia due to a differentiation block in erythroid precursors. The disease is commonly caused by mutations in genes for ribosomal proteins. Despite the identification of disease causal genes, the disease pathogenesis is not completely elucidated. The ribosomal abnormalities are assumed to inhibit globin translation which may lead to excess free heme, stimulating a generation of free radicals and thereby damaging the precursors. We studied the effect of hemin (heme chloride) on cultured human erythroid precursors and found that contrary to aforementioned hypothesis, although hemin moderately stimulated free radicals, it did not cause apoptosis or necrosis. In erythroid precursors derived from DBA patients, hemin significantly stimulated growth and hemoglobinization. Thus, heme toxicity is unlikely to play a role in the pathophysiology of most DBA cases. Moreover, its beneficial effect in culture suggests a therapeutic potential.

## 1. Introduction

DBA is a rare congenital form of pure red-cell anemia, characterized by macrocytic anemia, reticulocytopenia, and a block in erythroid differentiation at the proerythroblast stage, often in association with physical anomalies and growth retardation [1]. A large proportion of the patients carry mutations in genes encoding for ribosomal proteins, for example, RPS19, RPS24, and RPS17 [1]. Based on this genetic information, defects in ribosomal biogenesis are postulated to underlie the pathogenesis of the disease [1]. Although gross defects in ribosomal function are not compatible with viability, subtle defects may exhibit tissue specificity and impair only certain cellular functions where the requirement for high-level translation for a particular critical gene product is not fulfilled. In DBA, defects in ribosomal biogenesis are suspected to impair the initiation of globin translation, causing a mismatch between intracellular levels of globin chains and heme, a balance tightly coordinated under normal conditions. It has been suggested that a transient excess of heme is toxic to erythroid precursors via iron-mediated generation of free radicals [2].

Heme, however, is not invariably toxic to developing erythroid precursors (or to other cells) [3]. Exogenously supplied hemin **(**heme chloride) is readily taken up by cultured erythroid cells [4], and its iron is incorporated into hemoglobin (Hb) or stored in ferritin [5]. Following the addition of succinylacetone, a potent inhibitor of heme synthesis, exogenously supplied hemin was shown to substitute for intracellularly synthesized heme and to incorporate into *de novo* formed Hb [5]. Several groups reported that hemin supplementation to semisolid cultures promotes the growth of normal erythroid precursors, (e.g., [6]). We previously showed, in a two-phase liquid culture, that hemin promotes normal erythropoiesis by accelerating the proliferation and hemoglobinization of erythroid precursors in the presence [7] or absence [8] of holotransferrin. This effect was particularly prominent during the early stages of maturation, when iron-uptake and heme synthesis are the rate-limiting steps of hemoglobinization.

We now report that although hemin at subtoxic concentrations moderately stimulates free radical generation in erythroid precursors, it does not cause apoptosis or necrosis. In DBA erythroid precursors, hemin partially restores the growth and differentiation potential. The beneficial effect of hemin on these precursors may be related to its ability to supply heme at early stages of hemoglobinization, when heme synthesis is low, and thus overcome (at least partly) the inhibitory effect of their abnormal ribosomes. This suggests that, contrary to what has previously been suggested [2], heme excess does not play a role in the pathogenesis of most cases of DBA. Furthermore, our data suggest that hemin might be of therapeutic potential in DBA and other disorders with hematopoietic abnormalities associated with ribosomal dysfunction.

## 2. Patients and Methods

The research was approved by the Hadassah—Hebrew University Medical Centre Human Experimentation Review Board. Peripheral blood samples were obtained from normal donors and patients that met the criteria of DBA [9]. Erythroid cells were cultured according to the two-phase liquid culture procedure as previously described [10]. In short, peripheral blood-derived mononuclear cells were first cultured in alpha medium supplemented with 10% fetal calf serum and 10% conditioned medium obtained from cultures of human bladder carcinoma cell line 5637 and 1 *μ*g/mL cyclosporin A (phase I). After 6 days, nonadherent cells were harvested, washed, and suspended in phase II medium, containing alpha medium, 30% fetal calf serum, 1% bovine serum albumin, 10 *μ*M *β*-mercaptoethanol, 1.5 mM glutamine, 10 *μ*M dexamethasone, 5 ng/mL stem cell factor, and 1 U/mL human recombinant erythropoietin. Hemin (bovine, Sigma, St. Louis, MO) was prepared as previously described [7]. Intracellular Hb was quantified by HPLC as previously described [7]. Hemogbloin-containing cells were scored microscopically following staining with benzidine dihydrochloride [10].

Reactive oxygen species (ROS), apoptosis, and necrosis were measured by staining with 2′-7′-dichlorofluorescin diacetate (DCF, Sigma), annexin-V, and propidium iodide, and the cells were analyzed by flow cytometry as previously described [11].

## 3. Results and Discussion

**Figure 1:**
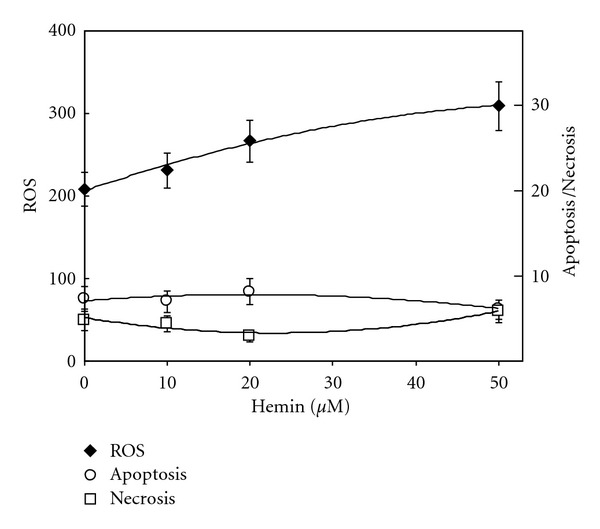
Effect of hemin on free radical generation, apoptosis, and necrosis of erythroid precursors. Mononuclear cells derived from the peripheral blood of normal donors were cultured according to the two-phase liquid culture procedure. After 6 days in phase II, hemin was added at the indicated concentrations for 16 hrs. Cells were harvested and stained with dichlorofluorescein diacetate, phycoerythrin-conjugated Annexin-V, or propidium iodide and analyzed by flow cytometry. The reactive oxygen species (ROS) data are presented as the mean fluorescence channel; apoptosis and necrosis data are presented as the percentage of positive cells. The results (average ± SD *N* = 4) show a modest dose-dependent stimulation of ROS by hemin but no effect on apoptosis and necrosis.

To study the potential toxic effect of hemin on developing erythroid precursors, normal erythroid precursors were cultured according to the two-phase liquid culture procedure [10]. After 6 days in phase II culture, hemin (10–50 *μ*M) was added for 16 hrs. The cells were then harvested and stained with DCF—a marker of free oxygen species (ROS), phycoerythrin-conjugated annexin-V—a marker of apoptosis which binds to phosphatidylserine exposed on the outer surface of the cells or propidium iodide—a marker of necrosis. The flow cytometry results (Figure 1) indicate that although ROS generation was modestly stimulated, it was not associated with apoptosis or necrosis.

**Figure 2:**
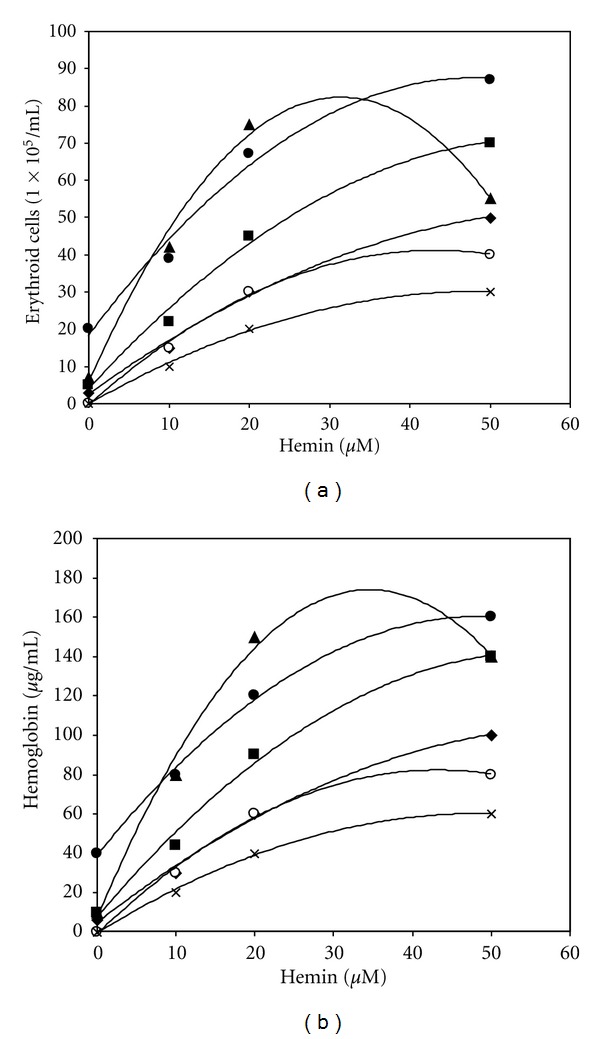
Effect of hemin on development of erythroid precursors derived from patients with Diamond-Blackfan Anemia. Peripheral blood mononuclear cells derived from 6 patients were cultured as in Figure 1. Hemin was added at the indicated concentrations on the first day of phase II. On day 12, hemoglobin-containing cells were enumerated microscopically following benzidine staining (a) and their intracellular hemoglobin content, by HPLC (b). The data represent the mean of duplicate cultures of cells derived from each patient. The results show significant (*P* < 0.001) stimulation by hemin of erythroid cell growth and hemoglobinization.

We next studied the effect of hemin in cultures of erythroid cells derived from patients with DBA. Hemin was added on the first day of phase II. The cells were then harvested on day 12. Hb-containing cells were enumerated by benzidine staining (Figure 2(a)) and their Hb content, by HPLC analysis (Figure 2(b)). In agreement with previous reports in semisolid cultures [12], very poor growth of DBA erythroid cells was observed in our liquid cultures. Hemin (10–50 *μ*M) significantly (4–20-fold, *P* < 0.001, and *N* = 6) increased their growth and hemoglobinization. Similar results were obtained when hemin was added as heme arginate (Leiras, Turku, Finland) (not shown).

Heme is involved in many metabolic pathways, including regulation of transcription through inhibition DNA-binding of the repressor, Bach1 [13]. In erythroid cells, it enhances globin translation through inhibition of the activity (substrate phosphorylation) of the repressor erythroid-specific “eukaryotic initiation factor 2*α* kinase” (eIF2*α* kinase) [14], it accelerates globin mRNA synthesis [15], and it stabilizes the newly synthesized globin chains by forming the Hb tetramer [16]. Normally, intracellular heme concentration is tightly controlled during erythroid differentiation: its synthesis is regulated by modulation of iron uptake through surface transferrin receptor-1 and the inducible 5-aminolevulinate synthase, the first enzyme of the heme synthetic pathway [17]. Moreover, heme excess is evaded by its degradation by heme oxygenase-1 [18] and its excretion by the heme export pump (Flvcr) [4]. Mice carrying a mutation in this pump present with a phenotype similar to human DBA [2]. The phenotypic similarity between the mouse model and the human disease led to hypothesis that heme excess may play a central role in the pathogenesis of DBA although no defect in this pump is found in patients [19].

Our data show that *in vitro* hemin within a particular concentration range is nontoxic to erythroid precursors, and, importantly, in DBA-derived cultures, it stimulates growth and hemoglobinization. These findings rule out the possibility that defective erythropoiesis in DBA is a consequence of intracellular accumulation of excess of heme. The beneficial effect of exogenous hemin on developing DBA erythroid precursors could be related to its ability to supply heme when heme synthesis is low and thus accelerate globin and Hb accumulation. This might overcome (at least partially) the inhibitory effect of their abnormal ribosomes, suggesting that hemin may be of therapeutic potential in DBA and other disorders with hematopoietic abnormalities associated with ribosomal dysfunction.
